# Results of a Prospective Randomized Multicenter Study Comparing Indocyanine Green (ICG) Fluorescence Combined with a Standard Tracer Versus ICG Alone for Sentinel Lymph Node Biopsy in Early Breast Cancer: The INFLUENCE Trial

**DOI:** 10.1245/s10434-024-16176-x

**Published:** 2024-09-12

**Authors:** Vassilis Pitsinis, Rahul Kanitkar, Alessio Vinci, Wen Ling Choong, John Benson

**Affiliations:** 1 Department of Breast Surgery, NHS Tayside, Dundee, UK; 2 Faculty of Medicine, University of Dundee, Dundee, UK; 3 Breast Unit, Cambridge University Hospitals NHS Foundation Trust, Cambridge, UK; 4 School of Medicine, University of Cambridge, Cambridge, UK; 5 Anglia Ruskin University, Cambridge, UK; 6 Edinburgh, UK

## Abstract

**Background:**

For clinically node-negative early breast cancer patients, sentinel lymph node biopsy (SLNB) using dual localization with blue dye and radioisotope (RI) is currently standard of care. Documented disadvantages with these tracers have prompted exploration of alternative agents such as fluorescent indocyanine green (ICG), which demonstrates high detection rates combined with other tracers. Results of a randomized study evaluating ICG as a single tracer for SLN identification are presented.

**Methods:**

Overall, 100 patients with unilateral, clinically node-negative, biopsy-proven invasive breast cancer (≤5 cm) scheduled for SLNB were recruited in two separate randomized cohorts, with 50 patients receiving ICG alone. Cohort 1 received ICG alone (*n* = 25) or combined with RI [Technetium^99^] (*n* = 25), while Cohort 2 received ICG alone (*n* = 25) or combined with blue dye (*n* = 25). The primary outcome was sensitivity for SLN identification.

**Results:**

Among evaluable patients (*n* = 97), the overall SLN identification rate was 96.9% (ICG alone = 97.9%; ICG + RI = 100%; ICG + blue dye = 92%). Node positivity rates were 14.9% for ICG alone, 16% for ICG combined with RI, and 20% for ICG combined with blue dye. There were no significant differences (*p* < 0.05) in performance parameters, with ICG alone being non-inferior to tracer combinations for procedural node positivity rates when adjusted for specific factors.

**Conclusion:**

These results support potential use of ICG as a sole tracer agent for routine SLNB, thereby avoiding disadvantages of RI and/or blue dye. The latter can be safely withheld as a co-tracer without compromising detection of positive nodes in primary surgical patients.

Staging of the axilla with sentinel lymph node biopsy (SLNB) at the time of breast surgery is the standard of care for clinically node-negative patients. Despite persistent variation in practice around the world, the current techniques for targeted sampling of SLNs in early-stage breast cancer is dual localization with blue dye and radioisotope (RI).^1^ Many breast units have opted to employ blue dye alone when radioactive tracers are unavailable or have abandoned the combination of blue dye with RI due to potential allergic reactions. Recent experience suggests that RI alone has comparable accuracy to a combined approach with blue dye. Nonetheless, use of radioactive tracers has some drawbacks, including rising material costs, supply issues consequent to contraction of the nuclear industry, radiation exposure of healthcare workers, and problems with surgical waste disposal. Furthermore, injection of radioactivity precedes the surgical procedure and requires a certain level of coordination between different disciplines to avoid any disruption to the patient care pathway that can be stressful to patients immediately before surgery. Alternative localization techniques using non-radioactive tracer agents such as indocyanine green (ICG), microbubbles, and superparamagnetic iron oxide nanoparticles have attracted attention over the past 20 years.^2–5^ The use of fluorescence navigation for visualization of lymphatic channels and nodal tissue in breast cancer patients has revealed high rates (>95%) of SLN identification.^6–11^

ICG provides at least equivalent detection rates and offers an additional dimension of real-time visualization of lymphatic tissue that RI localization lacks. Fluorescent tracers combine many advantages of blue dye and RI without the disadvantages of allergic reactions, staining of skin and surgical tissues, along with issues of handling/disposal of radioactive materials. Alternative technologies such as magnetic tracers pose challenges of interference relating to surgical instrumentation and breast imaging with MRI or with life-maintaining devices such as pacemakers. The combination of ICG with another tracer represents a transition phase and following accrual of clinical experience with usage in combination with other tracers, ICG is now being explored as a sole tracer agent. We present the results of a randomized study comparing ICG fluorescence combined with a standard tracer (blue dye or RI) versus ICG alone for SLNB in early-stage breast cancer patients. The aforementioned tracer combinations with dual localization are more expensive and inconvenient than ICG alone and are not necessarily associated with improved accuracy of a magnitude that is clinically meaningful.

## Materials and Methods

### Study Design and Patient Selection

In a prospective randomized study, 100 patients from the resident female population of Scotland and the East of England with unilateral clinically node-negative tumors scheduled to undergo routine SLNB for core biopsy-proven invasive breast cancer (<5 cm) were identified at multidisciplinary meetings. Male patients were excluded from the trial as they represented <1% of all breast cancer cases. Female patients aged at least 18 years were recruited from two centers: Cambridge University Hospitals NHS Foundation Trust (Cohort 1, *n* = 50) and NHS Tayside (Cohort 2, *n* = 50). Data were collected between 1 March and 31 December 2022 for 50 consecutive patients undergoing SLNB in each center. All patients had a normal preoperative axillary ultrasound and planned to undergo either breast-conserving surgery or mastectomy. Exclusion criteria included patients with non-invasive tumors, receipt of neoadjuvant chemotherapy, prior ipsilateral axillary surgery, or breast excision biopsy. Patients were also ineligible if pregnant, currently breastfeeding, or with any history of hypersensitivity to iodine or ICG.

Sentinel lymph node localization was undertaken with either single or dual tracer injection. Patients in Cohort 1 were assigned to ICG [2 mL 0.5%; 1 mL intradermal and 1 mL subareolar] alone (*n* = 25) or combined with RI [Technetium-99m nanocolloid, 20 MBq] (*n* = 25), whereas patients in Cohort 2 received ICG alone (*n* = 25) or combined with blue dye (2 mL 2.5% patent blue).

**Fig. 1:**
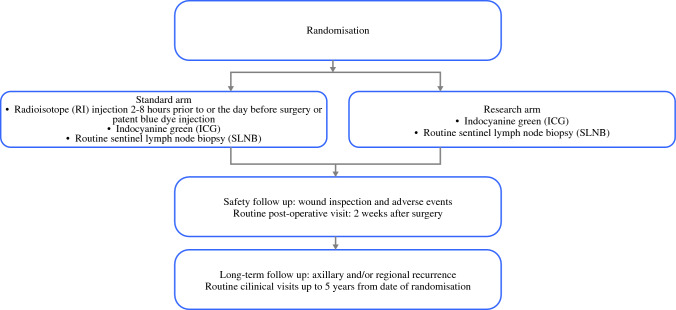
Study design

Patients had a follow-up clinic assessment at 2 weeks and a telephone consultation at 3 months following SLNB (±5 days) for recording of (1) concomitant medications; (2) any adverse skin reactions such as cutaneous staining, seroma formation, and other adverse reactions to tracers; and (3) any clinically diagnosed lymphedema (only in the 2-week follow-up period) (Fig. 1).

The trial was approved by the local NHS Tayside Medical Research and Ethics Committee (MREC).

### Sentinel Lymph Node Biopsy

In all cases, the SLNB procedure was performed by one of three surgeons (Cohort 1: JRB; Cohort 2: VP, AV), with fully informed consent being sought from all patients for trial participation. The breast was massaged for 2 min after injection of tracer (blue dye and/or ICG) at the edge of the areola at either the 10 o’clock (right breast) or 2 o’clock (left breast) position after induction of anesthesia.

**Fig. 2:**
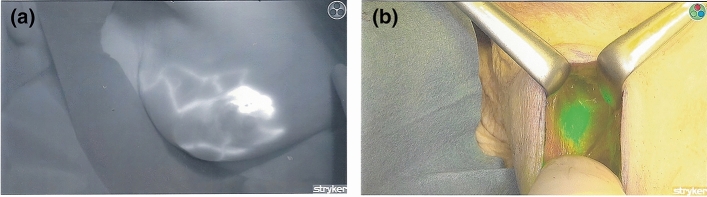
**a** Fluorescent image following periareolar injection of ICG in a right breast. ICG can be seen within the subcutaneous lymphatics coursing towards the axilla (camera on the black and white setting). **b** Fluorescent image of a sentinel lymph node with ICG as a single tracer (camera on green mode setting)

The lymphatic and nodal tissue were visualized with either a Photodynamic Eye Camera (Hamamatsu Photonics, Hamamatsu, Japan) or an infrared SPY-PHI camera (Stryker UK Ltd, Newbury, Berkshire, UK). Both consist of light-emitting diodes (LEDS), a lens, and a filter, with the detector being a charge-coupled device camera filtering out wavelengths below 820 nm. This technology relies on the generation of molecular fluorescence by ICG contact with plasma proteins in the lymphovascular system. This fluorochrome absorbs light at a wavelength of approximately 800 nm with emission of a fluorescent signal when subatomic particles return from an excited to ground state. The illuminated subcutaneous lymphatic channels can be seen on a photodynamic eye (PDE) camera display and ICG tracked as it passes towards the axilla (Fig. 2a). The fluorescent signal is captured by the PDE, which is composed of a series of LEDS, a lens, and a filter, with the detector being a charge-coupled device camera filtering out wavelengths below 820 nm. The fluorescent signal appears on a TV monitor and develops within seconds of peri-areolar ICG injection. The combination of ICG with either blue dye or RI allows both conventional and fluorescent modes for detection of nodes during SLNB. Once the skin incision is made, fluorescence provides navigation within the axillary space (Fig. 2b), although green lymphatic channels or nodes are sometimes apparent to the naked eye.

All excised nodes were examined ex vivo for fluorescence, with dissection of nodal tissue if necessary to ascertain the number of nodes retrieved macroscopically. The number of sentinel lymph nodes for each patient was recorded numerically and whether these were blue, radioactive, fluorescent, or a combination thereof. This permitted the calculation of nodal and procedural detection rates. The sensitivity of ICG alone and in combination with one or another standard tracer was calculated.

### Histopathology

Sentinel lymph nodes were serially sectioned at 2–3 mm intervals and subsequently stained with hematoxylin and eosin (H&E). Metastases were categorized as either macrometastases (>2 mm in size), micrometastases (>0.2 mm; ≤2 mm in size or <0.2 mm within the lymph node parenchyma), or isolated tumor cells (ITCs) [≤0.2 mm]. Immunohistochemistry was employed for those cases that were inconclusive on H&E staining.

### Statistics

Statistical analyses were performed using the Chi-square test or Fisher’s exact test with logistic regression to determine differences between the three groups. No interim analysis was considered appropriate due to rapid recruitment of patients and no adjustment for multiple testing was performed. The main objective was to assess non-inferiority of ICG alone compared with standard tracer combination. This was based on a confidence interval (CI) for the difference in percentage detection rate with a 5% non-inferiority margin.

## Results

### Patients

The mean age of patients was 65.1 years (standard deviation [SD] 10.72) with an average body mass index (BMI) of 28.2 (SD 5.51) for patients analyzed. There were marginally more screen-detected cancers (*n* = 50) than symptomatic cancers (*n* = 47), with three with missing patient data confined to the ICG-alone group (*n* = 22) in Cohort 1 (no missing patient data in Cohort 2) [see Table 1].

**Table 1:** Characteristics of patients in Cohorts 1 and 2 in combination

Variable	ICG alone [*n* = 47]	ICG + radioisotope [*n* = 25]	ICG + blue dye [*n* = 25]	Total
Age, years [mean (SD)]	64.4 (9.60)	65.7 (10.79)	65.6 (12.87)	65.1 (10.72)
Patient height, cm [mean (SD)]	162.6 (6.90)	160.8 (5.48)	159.8 (6.49)	161.4 (6.50)
Patient weight, kg [mean (SD)]	73.4 (13.45)	73.6 (20.02)	73.9 (16.08)	73.6 (15.86)
BMI, kg/m^2^ [mean (SD)]	27.8 (5.08)	28.2 (6.44)	28.8 (5.47)	28.2 (5.51)
Time to lymph node extraction, min [mean (SD)]	13.7 (9.82)	18.0 (8.91)	8.9 (4.34)	13.6 (9.03)
Ethnicity	Scottish patient population		East of England population	
	White 95%, Asian 3%, Mixed 2%		White 87%, Asian 6%, Black 3%, Mixed 3%, Arab 1%	

*ICG* indocyanine green, *SD* standard deviation, *BMI* body mass index

The mean tumor size for this mixed population of screen-detected and symptomatic cancers was 15.3 mm (range 5–45 mm). The majority of tumors were either invasive ductal carcinoma of no special type (NST) or invasive lobular carcinoma. The average nodal count was 2.4 for ICG alone (120/47), 3.1 for ICG combined with RI (78/25), and 1.9 for ICG combined with blue dye (47/25) [see Table 2].

**Table 2:** Breast cancer and sentinel lymph node biopsy data in Cohorts 1 and 2 in combination

Variable	ICG alone [*n* = 47]^a^	ICG + radioisotope [*n* = 25]	ICG + blue dye [*n* = 25]	Total
Breast cancer screen detected? [*n* (%)]	27 (54.0)^a^	17 (68.0)	6 (24.0)	50 (50.0)
Breast cancer palpable? [*n* (%)]	23 (46.0)^a^	14 (56.0)	18 (72.0)	55 (55.0)
Number of lymph nodes retrieved [mean (SD)]	2.4 (1.64)	3.1 (1.69)	1.9 (1.05)	2.5 (1.58)
Sentinel lymph node [*n/N* (%)]	82/120 (68.3)	55/78 (70.5)	43/47 (91.5)	180/245 (73.5)

*ICG* indocyanine green, *SD* standard deviation

^a^*n* = 3 excluded

A total of 100 patients were randomized between March and December 2022, with three patients excluded from analysis due to non-receipt of treatment allocation.

### Procedural Detection Rates

Among those patients available for analysis (*n* = 97), the overall SLN identification rate was 96.9%, and by tracer category was as follows: ICG alone = 97.9% (46/47); ICG and RI = 100% (25/25); ICG and blue dye = 92% (23/25) [see Table 3]. There were no significant differences (*p* > 0.05) in performance between ICG alone or in combination with one or another standard tracer. Therefore, ICG alone was non-inferior in terms of procedural detection rates, i.e. the proportion of cases in which an SLN(s) was successfully identified. Similar conclusions were reached from a secondary analysis adjusting for specific factors such as BMI, age, and mode of detection (screening vs. symptomatic).

**Table 3:** Procedural detection rates by tracer characteristics

Variable	ICG alone	ICG + radioisotope	ICG + blue dye	Total
Sentinel lymph node identified (%)^a^				
Yes	46 (97.9)	25 (100.0)	23 (92.0)	94 (94.0)
No	1 (2.1)	0 (0.0)	2 (8.0)	3 (3.1)
Total	47 (100.0)	25 (100.0)	25 (100.0)	97 (100.0)

*ICG* indocyanine green

^a^The percentage of success that the sentinel node has been identified by the different tracer modality. For example, under ICG alone, in 46/47 patients (97.9%), sentinel node(s) were successfully identified using ICG alone, as opposed to under ICG and radioisotope, where sentinel nodes were successfully identified in 25/25 patients (100%) using these techniques, and under ICG and blue dye, whereby sentinel nodes were successfully identified in 23/25 patients (92%) using these two techniques, therefore resulting in a total sentinel node identification rate of 94% among the three tracers used, as listed above

### Procedural Node Positivity Rates

The procedural node positivity rate is defined as the proportion of SLNB cases with tumor deposits in at least one node, including macrometastases, micrometastases, and ITCs. For Cohorts 1 and 2, the procedural node positivity rates were 14.9% for ICG alone, 16% for the combination of ICG with RI, and 20% for ICG combined with blue dye (see Table 4). There were no significant differences (*p* > 0.05) in the performance of ICG alone or combined with a standard tracer, with ICG alone being non-inferior in terms of procedural nodal positivity rates. Within Cohort 1, the node positivity rate for ICG alone was 17%, compared with 18% for ICG and RI. By contrast, there was some divergence in node positivity rates between ICG alone (12%) and ICG and blue dye (20%) within Cohort 2. Nonetheless, non-inferiority was maintained for both separate and combined analyses of ICG alone. Similar conclusions were reached from a secondary analysis adjusting for specific factors such as BMI, age, and mode of detection (screening vs. symptomatic).

**Table 4:** Procedural node positivity rates by tracer characteristics

Variable	ICG alone	ICG + radioisotope	ICG + blue dye	Total
At least one node identified containing metastases (%)				
Yes	7 (14.9)	4 (16.0)	5 (20.0)	16 (16.5)
No	40 (85.1)	21 (84.0)	20 (80.0)	81 (83.5)
Total	47 (100.0)	25 (100.0)	25 (100.0)	97 (100.0)

*ICG* indocyanine green

### Adverse Events

The principal adverse events that were evaluated were seroma formation, cutaneous staining, and requirement for seroma drainage. These events were assessed after 2 weeks and 3 months and are shown in Table 5.

**Table 5:** Adverse events by tracer

Characteristics	Time point	ICG alone [*n* = 47]	ICG + radioisotope [*n* = 25]	ICG + blue dye [*n* = 25]	Total [*n* = 97]
Cutaneous staining [*n* (%)]	2 weeks				
Yes		12 (26.1)	15 (60.0)	22 (88.0)	49 (51.0)
No		31 (67.4)	10 (40.0)	1 (4.0)	42 (43.8)
Cutaneous staining [*n* (%)]	3 months				
Yes		5 (11.6)	3 (12.0)	14 (56.0)	22 (23.7)
No		37 (86.0)	22 (88.0)	10 (40.0)	69 (74.2)

*ICG* indocyanine green

## Discussion

This prospective randomized study adds to the literature supporting the accuracy of utilizing the fluorochrome ICG as part of a dual localization strategy, and paves the way for a transition towards employment of this tracer as a single agent for SLNB in early breast cancer patients. Results confirm non-inferiority of using ICG alone compared with standard tracer combinations in terms of key performance indicators. Sentinel lymph node identification rates were high overall and by tracer category, with comparably high rates for combinations of ICG with either RI (100%) or blue dye (92%) and also for ICG alone (97.9%). The proportion of nodes retrieved that contained tracer and were fluorescent, radioactive, blue, or a combination thereof was similar for employment of ICG alone compared with a dual approach with no significant differences between tracer groups. Thus, nodal detection rates were 85.4% for ICG alone compared with 96.4% and 81.4% when combined with RI and blue dye, respectively. A crucial performance parameter is the overall node positivity rate and the ability of any tracer technique to detect those nodes containing tumor deposits. The overall node positivity rate with ICG alone was 14.9% which is consistent with a mixed population of screen-detected and symptomatic invasive cancers with a median tumor size of 15.3 mm.

The procedural node positivity rates within Cohort 1 were 17% for ICG alone and 18% for the combination of ICG and RI, while for Cohort 2, the node positivity rates diverged slightly for ICG alone (12%) and combined with blue dye (20%). Nonetheless there were no significant differences, and non-inferiority was maintained for separate and combined analyses of ICG alone. Furthermore, ICG alone was also found to be non-inferior to standard techniques for procedural positivity rates when adjusted for specific factors such as BMI and age.

The accuracy and safety of fluorescence imaging with ICG for SLNB in early breast cancer has consistently been demonstrated in published reports.^8–10,12^ Interestingly, early validation studies used ICG alone and cited identification rates in excess of 90%. Subsequent practice favored a combination of ICG with either blue dye or RI that allows both conventional and fluorescent visualization of lymphatic vessels and nodes. These have all confirmed high levels of nodal recognition by fluorescence, with few nodes (<5%) being classified as blue and/or radioactive but not fluorescent. A large multicenter Japanese study conducted by Sugie and colleagues employing a combination of ICG and blue dye for SLNB in breast cancer patients reported significantly superior identification rates for the fluorescent tracer compared with blue dye (99% vs. 78%; *p* < 0.001).^10^ These observations prompted a systematic review of eight studies comparing the relative performance of blue dye and ICG. It was concluded that ICG was significantly more accurate for SLN identification than the conventional tracer (odds ratio 18.37, 95% CI 8.83–39.10).^13^ One of the senior authors (JRB) published a feasibility study (ICG-10) to determine the sensitivity and safety of ICG in the context of routine SLNB using a triple tracer combination of blue dye, RI, and ICG. The sensitivity of ICG fluorescence was calculated as the percentage of SLNs detected by RI and/or blue dye that were also fluorescent. The SLN detection rates were 100% for ICG, 99% for blue dye, and 91.3% for RI.^12^ Moreover, the node positivity rate from this study was 17.3%, which accords with the rates observed in the current study. Other publications likewise confirm high sensitivity for ICG fluorescence in nodal detection in breast cancer cases.^9,10,12,14,15^ Moreover, studies evaluating the dual combination of ICG with either blue dye or RI have revealed high levels of concordance (>90%).^8,11,16^ A prospective observational study conducted by one of the authors (JRB) used a non-inferiority design to compare performance parameters of RI and ICG; the procedural detection rates were 97.5% and 98.7% respectively, with almost all nodes fluorescent (98.1%) and three-quarters (73.4%) radioactive.^9,15–17^

Despite much evidence testifying to the efficacy and favorable safety profile for ICG fluorescence, the majority of studies to date have relied on a dual tracer strategy involving the combination of ICG with a standard tracer for SLN detection. A combination of ICG with RI avoids potential adverse effects of blue dye such as allergic reactions and staining of the skin and breast tissue. The latter interferes with flap dissection at the time of skin- or nipple-sparing mastectomy, and cutaneous staining can last for up to 2 years. In the ALMANAC trial, the incidence of blue dye allergic reactions was estimated to be 0.1%.^18^ Following a total of 70 cases of allergic reactions to blue dye reported between 1975 and 2012, the Medicines Health and Regulatory Agency (MHRA) issued a drug safety update.^19^ Another study reported the overall incidence of the allergic reactions to isosulfan blue and patent blue V to be between 0.07 and 2.7%.^20^ In the current study, a single allergic reaction to blue dye was reported. Cutaneous staining with intradermal/subdermal injection of blue dye is observed in 70% of patients at 3 months and up to 40% at 1 year.^21^ These figures are similar to those reported herein, with cutaneous staining seen in 88% of cases at 2 weeks and 56% of cases at 3 months. By contrast, cutaneous staining attributable to ICG was less vivid and observed in 26% of cases at 2 weeks and only 11.6% of cases at 3 months. It should be noted that ICG has a short half-life and is rapidly excreted by the liver into the biliary system, thus providing an advantage over blue dye in terms of duration and intensity of cutaneous staining.^22^ Relative disadvantages of RI include radiation exposure of healthcare workers and patient inconvenience when an additional hospital visit is necessary for injection of RI the day before surgery (especially in breast reconstruction cases). A specific license for handling and disposal of radioactive material is mandatory and this precludes routine use of radioisotopes in many countries.^13^.

Fluorescence mapping with ICG is associated with high optical sensitivity and provides an additional dimension to SLN localization by allowing real-time visualization of subcutaneous lymphatic vessels and nodal tissues. This facilitates an orderly and stepwise dissection guided by fluorescent lymphatics that helps to identify sentinel nodes in order of ‘biological’ priority. With RI localization, SLNs are detected as hot spots, and not necessarily in the order of anatomical lymphatic flow. Moreover, with fluorescence navigation, those nodes containing metastases are more likely to be the first node removed.^11,12^

Nodal yield has been a controversial issue in the context of ICG fluorescence, with some concern expressed about an excessive number of nodes being fluorescent. Earlier studies reported nodal counts of between 3 and 5, which might potentially lead to increased morbidity from the SLNB procedure.^13,23,24^ However, more recent studies have not upheld these concerns, with average nodal counts of <2 per patient being frequently reported.^9^ In the ICG-10 study, the mean nodal count was 2.3 for the triple combination of ICG, RI, and blue dye,^14^ while the Milan group reported an average nodal count of 1.8 for the combination of ICG and RI, which is similar to a value of 2 for the same tracer combination.^9^ These nodal counts are also concordant with a study using ICG and blue dye (1.7) published by the two senior authors (VP, JRB). The current study found average nodal counts of 2.4 for ICG alone, 3.1 for ICG combined with RI, and 1.9 for ICG combined with blue dye. Although the average count is between 2 and 3, this is arguably a sensible number of nodes in an era of axillary de-escalation where a nodal count of 4 could be considered optimal for ensuring a low false negative rate (especially in the context of SLNB after neoadjuvant chemotherapy for node-positive disease at presentation).^11^

Obesity can adversely affect rates of SLN identification, with some studies noting significant attrition in rates among patients with a high BMI.^25,26^ Thus, obesity was correlated with SLNB failure within the ALMANAC trial, and Lin and colleagues reported identification rates of only 75% and 40% in patients with higher indices of 30 and 40, respectively.^25^ Blue dye-stained nodes are readily obscured by a modest layer of fatty tissue encasing the lymph nodes, but can often be otherwise visualized with fluorescence after opening up the axillary tissues at surgery. However, there is limited tissue penetration to a depth of 1.5–2 cm and this could therefore hinder visualization of subcutaneous lymphatics in obese patients.^11^ Nonetheless, a recent study examined nodal detection rates with ICG in 231 patients across a range of BMI values and showed no significant differences in uptake between various BMI categories (16–24.9, 25–29.9, 30–34.9 and ≥35) [*p* = 0.98].^27^ The average BMI for patients in the current study was 28.2 (SD 5.51), with the nodal detection rate for ICG alone being non-inferior to a dual tracer combination.

Tracer combinations with dual localization are more expensive and inconvenient than ICG alone, but are not necessarily associated with enhanced accuracy that translates into improved clinical outcomes. The current study provides further data supporting the safety and efficacy of ICG alone for SLNB in early breast cancer patients. In our setting, ICG has proven to be less expensive than RI tracers although initial capital equipment costs are higher. However, fluorescent devices have multiple clinical applications and are not confined to the setting of SLNB for breast cancer patients. In the longer term, use of ICG is likely to be more cost effective with lower costs per episode of care due to the relatively high costs of RI injection per patient (including materials and personnel).^28^ Use of ICG as a sole tracer agent can avoid the disadvantages of both radioisotopes (waste disposal, monitoring and mandatory licensing) and blue dye (cutaneous staining and allergic/anaphylactic reactions). These INFLUENCE trial results provide the first randomized data supporting the safety and efficacy of ICG alone for SLNB in early breast cancer patients and adds to accumulating evidence from observational studies that either blue dye or RI can be omitted as a co-tracer with reliance exclusively on ICG for sentinel node localization and detection of pathologically positive nodes in primary surgical patients.
